# Prescription of cardioprotective drugs after acute kidney injury

**DOI:** 10.1186/2197-425X-3-S1-A632

**Published:** 2015-10-01

**Authors:** A El-Solia, M Varrier, L Tovey, A Jones, M Ostermann

**Affiliations:** Guy's & St Thomas Hospital, London, United Kingdom

## Introduction

It is estimated that 5% of critically ill patients with acute kidney injury (AKI) require renal replacement therapy (RRT). Increasing data suggests that they have an increased risk of progressive chronic kidney disease, cardiovascular morbidity and premature death. The exact reasons for these poor outcomes following AKI are incompletely understood. Discontinuation of cardioprotective drugs may be an important factor.

## Objectives

To describe the influence of AKI requiring RRT on long term cardioprotective drug prescription in survivors to hospital discharge during a two year period.

## Methods

We identified all patients who received RRT for AKI in a large tertiary Intensive Care Unit (ICU) over a two year period. For those who recovered to RRT independence and survived to hospital discharge, documentation at admission and discharge from ICU and the hospital were reviewed to assess changes to medications and the information conveyed to primary care. We focussed on the following drugs: Metformin, renin-angiotensin-system blockers, beta-blockers, K sparing diuretics, statins and antiplatelet agents. Where patients were repatriated to other hospitals letters were sent to the general practitioners.

## Results

Full data was available for 130/178 patients (75%). Of 88 patients treated with at least one of the medications of interest 53 (60.2%) had at least one drug discontinued. (Table 1) Information regarding the AKI episode and the discontinued drugs were appropriately documented in the hospital discharge letters in only half of these.

**Table 1:** Table 1

		Patients on cardioprotective drugs pre admission				
CKD stage at hospital discharge as per eGFR	Number of patients with severe AKI who survived to hospital discharge	Number of patients	Mean baseline eGFR pre-hospitalisation (when available)	Mean eGFR at hospital discharge	Number of patients in whom cardioprotective drugs were discontinued and not restarted after hospital discharge	Appropriate documentation in hospital discharge letter regarding AKI and drug discontinuation (% of relevant patients)
**V**	3 (2.3%)	2	>60 (2 pts)	12	2 (100%)	2 (100%)
**IV**	18 (13.8%)	14	38.9 (9 pts)	24.1	9 (64%)	5 (55.5%)
**IIIb**	28 (21.5%)	25	59.4 (21 pts)	39	17 (68%)	12 (71%)
**IIIa**	24 (18%)	15	44.1 (7 pts)	52.4	8 (53%)	3 (38%)
**I or II**	58 (44.6%)	32	>60 (22 pts)	>60	17 (53%)	8 (47%)
**Total**	130	88	61 pts	88 pts	53 (60.2%)	30 (56.6%)

Abbreviations: AKI = acute kidney injury; eGFR = estimated glomerular filtration rate; CKD = chronic kidney disease.

## Conclusions

1. It was common for patients with AKI to be prescribed cardioprotective drugs at the time of admission and for some or all to be discontinued during critical illness. Many patients recovered a good level of renal function by hospital discharge but still had medications discontinued. Whilst it may have been appropriate during the acute illness, prolonged discontinuation may have had an effect on the stability of co-morbid disease in the longer term.

2. Although primary care physicians are increasingly responsible for management of chronic conditions and rely upon accurate and complete information from secondary care, the transfer of relevant information related to medication management was frequently sub-optimal

## Grant Acknowledgment

The work was undertaken during an ERA-EDTA Young Fellowship.

